# Complete Genome Sequence of Bifidobacterium dentium Strain JCM 1195^T^, Isolated from Human Dental Caries

**DOI:** 10.1128/genomeA.00284-15

**Published:** 2015-04-09

**Authors:** Hidehiro Toh, Jun-ichiro Hayashi, Kenshiro Oshima, Akiko Nakano, Yukiko Takayama, Kageyasu Takanashi, Hidetoshi Morita, Masahira Hattori

**Affiliations:** a Medical Institute of Bioregulation, Kyushu University, Fukuoka, Japan; b School of Dentistry, Aichi Gakuin University, Nagoya, Japan; c Graduate School of Frontier Sciences, University of Tokyo, Kashiwa, Japan; d Department of Microbiology and Immunology, Teikyo University School of Medicine, Tokyo, Japan; e School of Veterinary Medicine, Azabu University, Sagamihara, Japan

## Abstract

Bifidobacterium dentium strain JCM 1195^T^ was isolated from human dental caries. Here, we report the complete genome sequence of this organism.

## GENOME ANNOUNCEMENT

Bifidobacterium is frequently isolated from the human intestine, but Bifidobacterium dentium is known to be present in the human oral cavity, as well as in the intestine. B. dentium is frequently isolated from active carious lesions and thus may contribute to the pathogenesis of dental caries. Possession of an enzyme activity that degrades artificial trypsin substrates, such as benzoylarginine-β-naphthylamide, has been proposed as a possible virulence factor in the suspected periodontal pathogens (1, 2). B. dentium, which is one of the periodontal bacterial isolates, also has this activity (3). B. dentium belongs to the Bifidobacterium adolescentis group (4).

B. dentium strain JCM 1195^T^ (DSM 20436^T^) was isolated from human dental caries (5). We determined the complete genome sequence of B. dentium JCM 1195^T^ using a whole-genome shotgun strategy with Sanger sequencing (ABI 3730xl sequencers). We constructed small-insert (2-kb) and large-insert (10-kb) genomic DNA libraries and generated 33,024 sequence reads (9.3-fold coverage) for B. dentium JCM 1195^T^ from both ends of the genomic clones. Data were assembled with the Phred-Phrap-Consed program. Gap closing and resequencing of low-quality regions were conducted by Sanger sequencing to obtain the high-quality finished sequence. The overall accuracy of the finished sequence was estimated to have an error rate of <1 per 10,000 bases (Phrap score of ≥40). An initial set of predicted protein-coding genes was identified using Glimmer version 3.0 (6). Genes consisting of <120 bp and those containing overlaps were eliminated. The tRNA genes were predicted by tRNAscan-SE (7), and the rRNA genes were detected by a BLASTn search using known Bifidobacterium rRNA sequences as queries.

The genome sequence of B. dentium JCM 1195^T^ consists of a circular chromosome of 2,635,669 bp with no plasmid. The genome size is larger than those of the other species in the B. adolescentis group, such as B. adolescentis, B. angulatum, B. catenulatum, and B. pseudocatenulatum. JCM 1195^T^ contained a clustered regularly interspaced short palindromic repeats (CRISPR) (8) region (1,831,394 to 1,836,771), and five CRISPR-associated genes (BBDE_1555 to BBDE_1559) were encoded upstream of the CRISPR region. The chromosome contained 2,141 predicted protein-coding genes, 2,066 (97%) of which were conserved in the genome of B. dentium Bd1 (9). JCM 1195^T^ contained seven pilus gene clusters, all of which also were found in the genome of B. dentium Bd1 (10). Of the 2,141 protein-coding genes, 1,307 (61%) were conserved in the genome of B. adolescentis ATCC 15703^T^ (accession no. AP009256). The remaining 834 genes contained nine carbohydrate utilization gene clusters, which consist of a carbohydrate transporter, glycosyl hydrolase, and transcriptional regulator (BBDE_0114–BBDE_0129, BBDE_0465–BBDE_0469, BBDE_0627–BBDE_0633, BBDE_1007–BBDE_1010, BBDE_1208–BBDE_1212, BBDE_1611–BBDE_1616, BBDE_1980–BBDE_1986, BBDE_1996–BBDE_2043, and BBDE_2051–BBDE_2055). The genome information of this species will be useful for further studies of its physiology, taxonomy, and ecology.

### Nucleotide sequence accession number.

The sequence data for the genome have been deposited in DDBJ/GenBank/EMBL under the accession number AP012326.
